# Prevalence of sweetpotato viruses in Acholi sub-region, northern Uganda^☆^

**DOI:** 10.1016/j.cpb.2019.03.001

**Published:** 2019-01

**Authors:** Godfrey Wokorach, Hilary Edema, Dennis Muhanguzi, Richard Echodu

**Affiliations:** a Biosciences Research Laboratory, Gulu University, P.O. Box 166, Gulu, Uganda; b Department of Biomolecular Resources and Biolaboratory Sciences, School of Biosecurity, Biotechnical and Laboratory Sciences, College of Veterinary Medicine, Animal Resources, and Biosecurity, Makerere University, P.O. Box 7062, Kampala, Uganda; c Department of Biology, Faculty of Science, Gulu University, P.O. Box 166, Gulu, Uganda

**Keywords:** Sweetpotato mild mottle virus, Sweetpotato chlorotic fleck virus, Chlorotic fleck virus, Northern Uganda

## Abstract

The purpose of the study was to identify different viruses infecting sweetpotato and the level of co-infection and spatial distribution of the viruses within the Acholi sub-region of northern Uganda. Multiplex PCR was used to screen and determine level of co-infection in 380 sweetpotato plants. The PCR scores were computed to give overall frequency of occurrence of different viruses. The spatial distribution of viruses was represented on an ArcGIS map. Of all screened samples, 24% (92/380) were infected with at least one virus. Sweetpotato feathery mottle virus (65/92), sweetpotato chlorotic fleck virus (17/92) and sweetpotato mild mottle virus (8/92) were the most frequent viruses detected. Of sampled fields, 74% (28/38) had at least one virus-infected sweetpotato plant. The four viruses detected are the major viruses causing significant yield losses in major sweetpotato growing regions of Uganda and East Africa. The findings of limited distribution and low prevalence of the viruses in the region indicate it causes less burden to sweetpotato production in the sub-region compared with other parts of Uganda.

## Highlights

• Four sweetpotato viruses were confirmed to infect sweetpotato plants in northern Uganda.
• Sweetpotato feathery mottle virus had the highest prevalence compared to other viruses detected.
• Kitgum district had the highest number of infected sweetpotato plants compared to Gulu and Lamwo districts.

## 1 Introduction

Sweetpotato is an important crop for smallholder farmers in resource-limited rural settings of Africa. It requires few inputs to grow, yields relatively well in poor soils and is drought tolerant [1]. It is a good carbohydrate source and the cheapest food security crop for subsistence farmers in Africa [2,3]. In addition, sweetpotato tubers and leaves are regarded as the cheapest source of vitamins (A, riboflavin, thiamine and niacin), micro-nutrients (iron, zinc, calcium and magnesium), protein, fat and dietary fibre [[3], [4], [5]]. The importance of sweetpotato is constantly increasing but its production is greatly constrained by viruses, among other biotic factors. Up to seven sweetpotato viruses have been reported to infect and constrain sweetpotato production in East Africa. Six of these have been particularly reported in Uganda [6,7], where they can cause up to 98% yield losses [8].

Propagation of sweetpotato plants using vine cuttings remain the most important mechanisms for the spread, survival and transmission of sweetpotato viruses from generation to generation [9]. In addition, traditional agricultural practices such as piecemeal harvest allow the virus to be maintain for long within the infected plants such that it act as potential source of inoculum for future infection [1]. Sharing of sweet potato vines amongst farmers or buying vines from the market during time of shortages are some of the farming practices that promote the spread of sweetpotato viruses among different farms [10]. SPCSV is transmitted by whitefly common species known as *Bemisia tabaci* while SPFMV is transmitted by aphids (*Aphis gossypii*) [10,11]. Some of the viruses are transmitted through sap inoculation from infected plant by use of contaminated tools during vine cutting among the local farmers [12].

Most sweetpotato viruses do not produce severe symptoms as single infections but have devastating co-infection effects [9]. Synergistic interaction among sweetpotato feathery mottle virus (SPFMV), sweetpotato mild mottle virus (SPMMV) and sweetpotato chlorotic stunt virus (SPCSV) causes a very severe sweetpotato condition – sweetpotato chlorotic dwarf disease [10]. Co-infections involving SPFMV and SPCSV produce a severe disease syndrome known as sweetpotato virus disease (SPVD) that is associated with severe yield losses in a number of sweetpotato production systems [1,11,12]. Currently SPVD is widespread in the major sweetpotato growing region of Uganda [6] and has been implicated in elimination of some early maturing and high yielding cultivars [11]. In addition, high incidences of SPFMV and SPCSV have been reported in central Uganda [6,8]. However, reports on the incidence of these viruses and their effect on sweetpotato production in the former war-zone of northern Uganda are limited. Such information is essential in guiding control strategies toward managing spread of these diseases. This study was therefore carried out to determine the prevalence of different sweetpotato viruses in northern Uganda.

## 2 Materials and methods

### 2.1 Study area

**Fig. 1:**
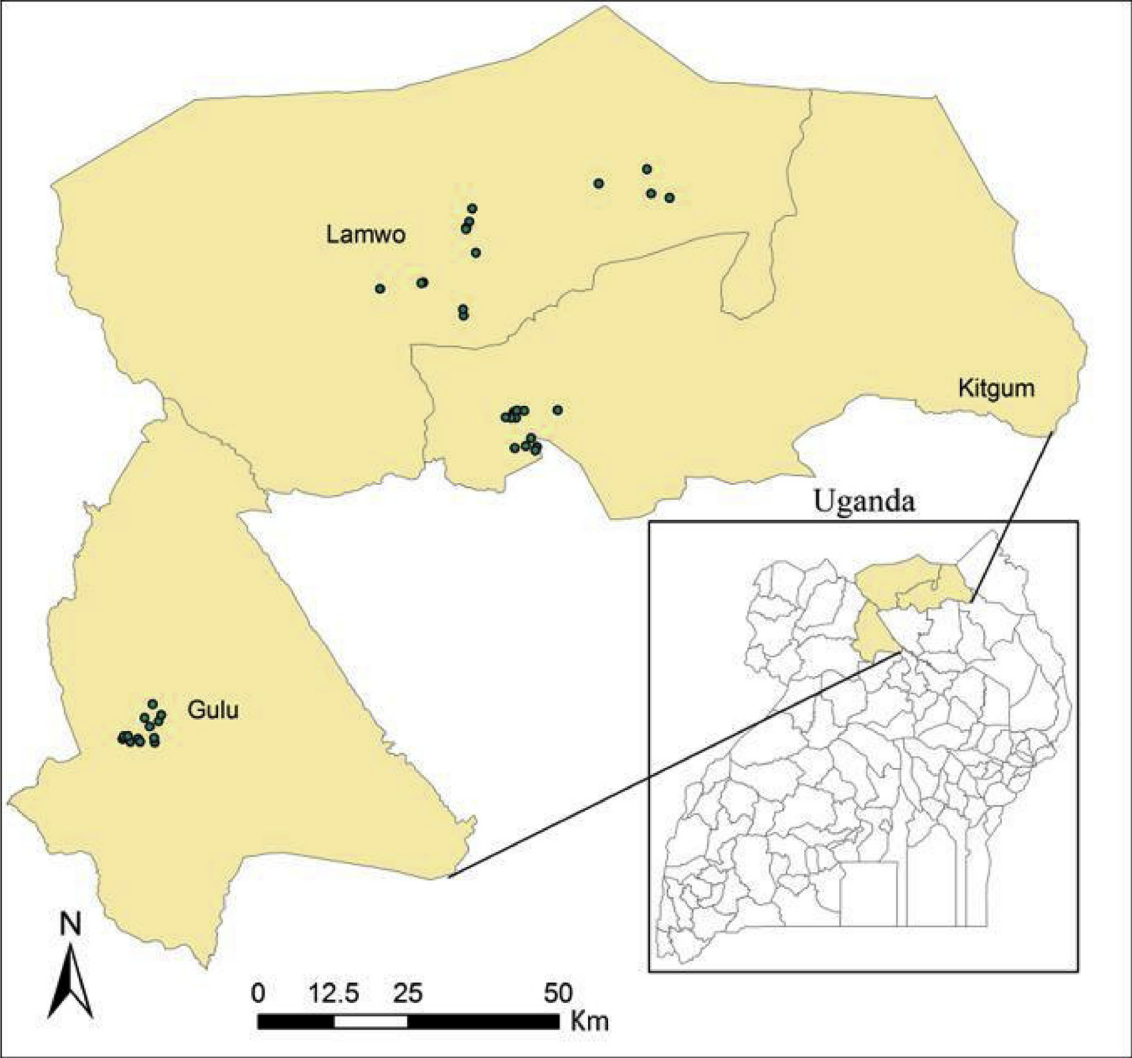
Map showing location of the study site. Green dots represent the sampled sweet potato fields.

A cross-sectional survey was carried out in Gulu, Kitgum and Lamwo districts of the Acholi sub-region in northern Uganda (Fig. 1) from January to February 2016. These districts were chosen because they represent the major sweetpotato growing districts in Acholi. A total of 380 samples were collected from 38 fields across six sub-counties randomly selected from the three districts.

### 2.2 Sweetpotato field and vine sampling strategy

Sweetpotato fields were sampled using systematic random sampling along roads [13,14]. The distance between a sampled field and a subsequently sampled field was at least 2 km [15]. Only fields with vines aged two months or more were sampled because they had developed many leaves for symptom observation. Field observations were made to identify vines with symptoms related to virus infection [16]. The picture of the plant showing symptom of viral infection were taken from the field and vines were cut at least 15 cm long. Leaves were removed from the vines and subsequently the vines were wrapped in moist tissue paper to avoid withering. The sampled vines were potted in a screen-house at Gulu University a day after their collection from the fields [15,16]. New leaves were monitored for any development of virus like symptoms similar to those manifested by the plant when in the field to differentiate symptoms induced by heat stress or insect bites when the plant where in the fields. The vines were watered regularly every two days and also sprayed with insecticide to avoid cross infection by insect vectors [17]. Leaves of the plant were harvested within three to four weeks after potting for testing virus infection.

### 2.3 Molecular typing of sweetpotato viruses

Viral RNA or DNA was extracted using TRIzol LS reagent (Invitrogen, Carlsbad, CA, USA) from fresh leaves of sweetpotato plants established in the screen-house. The RNA quality was checked by denaturation in highly deionised HI-DI^™^formamide (Thermofisher Scientific, Waltham, MA, USA) and electrophoresed in 1.2% agarose dissolved in 1% TAE buffer [18].

The cDNA was generated using a RT-PCR kit (New England Biolabs Inc., Ipswich, MA, USA). The reaction volume contained 10μM of 0.5 μl of each reverse primer, 200000U/ml of 0.5 μl M-MuLVR reverse transcriptase, 1X of 5 μl of RT buffer, 1 mM of 4 μl of dNTPs mix, 40000U/ml of 1 μl of RNase inhibitor, 2 μl of RNA template, 10 μg/ml of 0.5 μl of BSA and water to bring the total reaction volume to 20 μl. The reactions were then incubated in SimpliAmp Thermal Cycler (Life Technologies, Marsiling Industrial Estate Road3, Singapore) under the following conditions: 22 °C for 10 min, 42 °C for 40 min and 95 °C for 4 min.

**Fig. 2:**
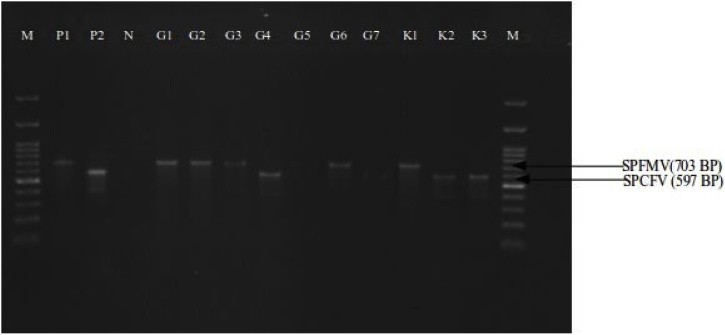
Gel image showing PCR results. M was 100 BP DNA ladder. P1 and P2 were positive controls for SPFMV and SPCFV. N is negative control. G series were samples from Gulu district and K series were samples from Kitgum districts.

Multiplex PCR was completed in a 25 μl reaction volume using a *Taq* PCR kit (New England Biolabs Inc.). The reaction mixed contained 1X of 5 μl of PCR buffer, 1 mM of 2 μl of dNTP solution mix, 5000U/ml of 0.2 μl of *Taq* polymerase, 2.5 mg/ul of 4 μl of MgCl_2_, 2 μl of cDNA templates, 10μM of 0.5 μl of forward primers, 10μM of 0.5 μl of reverse primers and PCR water to make the volume up to 25 μl. Amplification was performed in SimpliAmp Thermal Cycler as follows: initial denaturation at 94 °C for 5 min and 35 thermal cycles of denaturation at 94 °C for 30 s, annealing at 50 °C for 30 s, extension at 72 °C for 1 min. Final extension was at 72 °C for 5 min. The PCR products were electrophoresed in 1% Agarose Basic (AppliChem GmbH, Darmstadt, Germany), stained with SYBR Safe DNA gel stain (Invitrogen) and visualised on an UVIDOC HD5 (UVITEC, Cambridge, UK) ultraviolet trans-illuminator. Infections were determined when one or more bands corresponding to expected amplicon sizes (Table 1 and Fig. 2) of the viruses appeared on the lane in agarose gel after electrophoresis.

**Table 1:** Primers list used for the PCR.

Virus	Primer name	Primer sequence (5’-3’)	Fragment Size	Reference
SPCSV	Cp1 (forward)	CTG CTA GAT TGT TAG AAA	1150BP	[25]
	Cp2 (reverse)	TAT ATG AAA ATA TAG TTC		
SPFMV	SPFMV-F	GGACGAGACACTAGCAA	703BP	[22]
	SPFMV-R	TTCTTCTTGCGTGGAGACGT		
SPMMV	MMA1 (forward)	CCATTCAGAACAAGGAGC	117BP	[23]
	MMD2(reverse)	TTGAGCTCCTCTCAGACT		
SPCaLV	F2(4)	AGGAAATCCCAGTATTATTCAAC	922BP	[24]
	R2(4)	ATTTCTAATTTGGTTTACTAATCC		
SPCFV	SPCFV 2F	AGCTGCTCAAACAAGCAAGAGG	597BP	[20]
	SPCFV 2R	GCTCAAAAGTACTTTAAAACATGC		
SPLCV (Begomovirus)	SPG3	ACTTCGAGACAGCTATCGTGCC	1148BP	[7]
	SPG4	AGC ATG GAT TCA CGC ACAGG		

Sweet potato chlorotic stunt virus (SPCSV), Sweet potato feathery mottle virus (SPFMV), Sweet potato mild mottle virus (SPMMV), sweet potato caulimo like virus (SPCaLV), sweet potato chlorotic fleck virus (SPCFV), sweet potato leaf curl virus (SPLCV).

### 2.4 Data analysis

The gel electrophoresis bands were used to summarised virus infection of the samples after PCR. Samples with gel electrophoresis band corresponding to expected amplicon size were recorded as positive in Excel spread sheet. Those with no bands were recorded as negative for the virus infection in the Excel spread sheet. The data recorded in excel spread sheet were uploaded to EPI info 7 (CDC, Atlanta, USA). The frequency of all infections was calculated and expressed as a percentage. Similarly, frequency of infection of sweetpotato fields were computed and expressed as percentages. The 95% confidence interval for percentage infection was calculated. The spatial distribution of the different viruses across the three districts within Acholi were presented in an ArcGIS map.

## 3 Results

### 3.1 Sweetpotato viruses detected in Acholi

Only four viruses (SPFMV, SPMMV, SPCFV and SPCSV) were detected in Acholi (Table 2). A total of 92/380 (24.2%) samples were infected with any of the four viruses. Of these 92 infections, 65/92 (70.65%) were SPFMV, which represented the major virus infecting sweetpotato in the region (Table 2). There were 17/92 (18.5%) infections with SPCFV, 8/92 (8.70%) of SPMMV and only 2/98 (2.20%) were SPCSV. In total, 17.11% (65/380) of samples were infected with SPFMV, 4.47% (17/380) with SPCFV, 2.11% (8/380) with SPMMV and only 0.5% (2/380) with SPCSV.

**Table 2:** Viruses infecting sweetpotato in the Acholi sub-region, n = 380.

Virus	Frequency	Percent	95% LCL	95% UCL
SPCFV	17	4.47	2.81%	7.05%
SPCSV	2	0.53	0.14%	1.90%
SPFMV	65	17.11	13.65%	21.22%
SPMMV	8	2.11	1.07%	4.10%
No infection	288	75.79	71.24%	79.82%

LCL is lower confident limit and UCL is upper confident limit.

### 3.2 Spatial distribution of sweetpotato viruses detected in Acholi

**Fig. 3:**
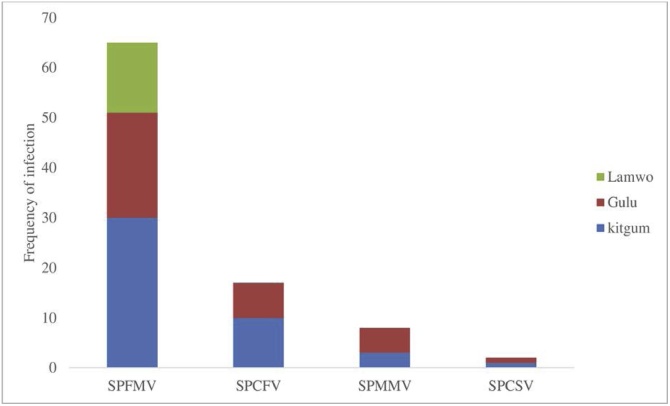
Prevalence of SPCFV, SPCSV, SPFMV and SPMMV infection within the three districts.

The highest number of virus-infected samples (44/380) was from Kitgum, followed by Gulu (34/380) and then Lamwo (14/380). Total SPFMV infection in Kitgum was 30/44, SPCFV was 10/44, SPMMV was 3/44 and SPCSV was 1/34 (Fig. 3). Similarly, in Gulu, 21/34 samples were infected with SPFMV, 7/34 with SPCFV, 5/34 with SPMMV and 1/34 with SPCSV. Only SPFMV was detected in Lamwo.

**Fig. 4:**
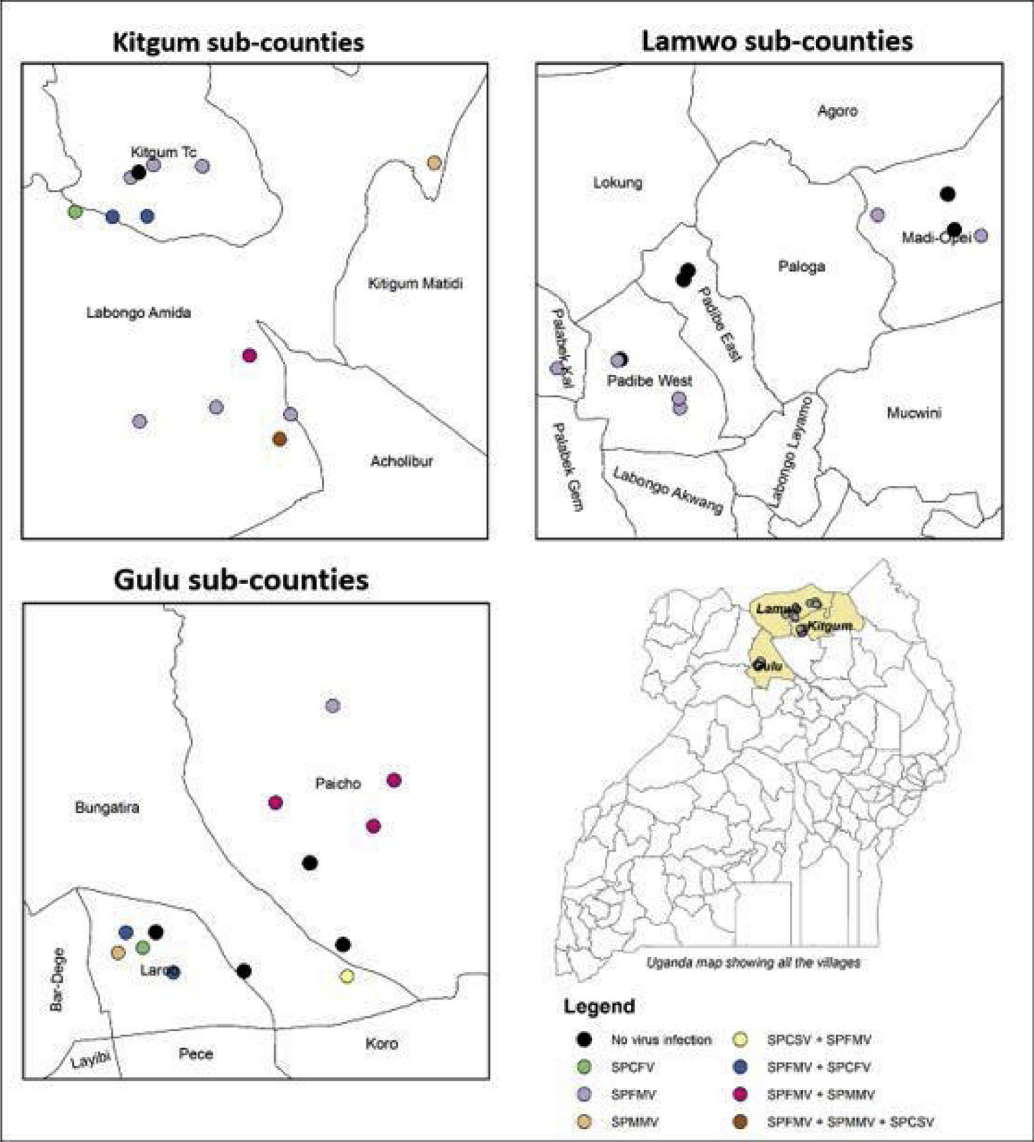
Spatial distribution of SPCFV, SPCSV, SPFMV and SPMMV within sweetpotato fields in the three districts.

No virus was detected in 26% (10/38) of surveyed fields, SPFMV occurred in 57.9% (22/38) of surveyed fields, SPMMV occurred in 18.4% (7/38), six (15.8%) had SPCFV and only two (5.3%) had SPCSV. Five fields had both SPFMV and SPMMV, four had infection with both SPFMV and SPCFV, and two fields had both SPCSV and SPFMV (Fig. 4). Only one field had both SPFMV, SPCSV and SPMMV (Fig. 4).

### 3.3 Co-occurrence of sweetpotato viruses detected by multiplex PCR

Only three samples out of 380 showed infection by more than one virus. Only two kinds of co-infection were identified: SPFMV + SPCFV (two samples, one each from Kitgum and Gulu) and SPCSV + SPCFV (one sample from Gulu). Co-infection of SPCSV and SPFMV was not detected in any of the three districts. Furthermore, multiple infection involving three or more viruses was not detected using multiplex PCR.

## 4 Discussion

The most widespread virus identified in the study was SPFMV (Table 2), with higher frequency of occurrence than other viruses. This finding is consistent with studies done in central, western and eastern Uganda that indicated wide distribution of SPFMV among farmers’ fields [6,8]. In different parts of East Africa, SPFMV remains the most widely distributed virus [13,15]. Reports also indicate that SPFMV occurs in almost every country where sweetpotato is grown [19]. The widespread distribution of SPFMV in different part of the world is attributed to its ability to cause mild or no symptoms in sweetpotato plants, making it difficult for farmers to detect SPFMV infection during vine propagation and so promoting its spread by sharing infected vines among local farmers [15]. As well the mild or no symptom manifested by SPFMV make it hard for farmers to rogue the infected sweetpotato plants from their sweetpotato fields. Such sweetpotato plant may be propagated and reuse for many seasons by different farmers through vine sharing which is a common practice of vine acquisition among local farmers. Single infection by SPFMV is estimated to cause yield losses of about 1.6% in sweetpotato. Some strains of the virus cause corkiness in root tubers making them unpalatable.

The SPCSV was least detected and had limited distribution (Table 2). However, reports indicate that it is the major virus causing significant yield losses in central and western Uganda where it often occurs in combination with SPFMV [6,8]. Limited Prevalence of SPCSV has been reported in some parts of Uganda and Tanzania [6,13]. SPCSV infected plant manifest symptom clearly which make some farmers select against such vines when selecting the vines for propagation. Phytosanitary measures such as roughing sweetpotato plants with virus like symptoms by sweetpotato farmers could contribute to low prevalence of SPCSV reported in this study since the virus clearly manifest symptom that are easily detected by farmers. The prevalence of SPCSV in different agro-ecological area also depends on the abundance and distribution of whitefly [23,24]. However, the hot dry season in northern Uganda from December to April every year scorch most sweetpotato vines in the area and provide break in whitefly lifecycle which is a key vector of SPCSV [12]. Despite its low prevalence, detection of SPCSV in this area poses a threat to sweetpotato production since it can cause an estimated yield loss of about 40% alone. However, up to 98% yield losses have occurred when SPCSV and SPFMV co-infect sweetpotato [8]. It is the major sweetpotato virus responsible for degeneration and extinction of sweetpotato cultivars [24]. Currently, local farmers often reuse and share planting materials and this will most likely increase the frequency of occurrence of SPCSV and other viruses.

The SPCFV was the second most detected virus in our study. A previous study ranked it as the fourth most important virus in central and western Uganda [6] and an earlier study indicated that SPCFV had comparatively higher prevalence in a surveyed district in northern Uganda [8]. No vector has yet been identified as responsible for transmission of SPCFV, making it impossible to correlate spread of SPCFV with a vector. The spread of SPCFV through sap from infected sweetpotato is the only known mode of transmission [20]. The high frequency of detection is possibly due to the sharing of infected planting material among local farmers and spread through sap inoculation from unsterilised tools during cutting of planting materials.

The SPMMV, a potyvirus, was the third most important virus detected in our study and had a lower frequency of detection compared with other studies. It is the third most distributed virus in Uganda and East Africa [6,8,21]. It can interact with SPFMV and SPCSV, producing the severe disease syndrome known as sweetpotato chlorotic dwarf disease as first reported in Argentina [10]. The three-virus combination has been reported in Uganda and shows severe disease syndromes with significant yield losses [6,8].

Infection involving two or more viruses was rare in our study and single infection by SPFMV was the most common, in contrast to findings in central and western Uganda where most infection was mixed [6,8]. Our findings were consistent with previous results [6] in which SPFMV was the major virus of sweetpotato in northern Uganda. Similarly, single infection by SPFMV was more common than multiple infection in Tanzania [13]. However, we failed to detect infection of SPCSV and SPFMV, which together form the common and devastating disease of SPVD in East Africa [13,15,22]. This is not the first case of unusually low occurrence of SPVD – a similar case was reported in the coastal district of Bagamoyo in Tanzania [13]. The possible explanation for the failure to detect SPVD is the rare occurrence of SPCSV in this region, as found in our study and previously [6].

Previous studies attributed the low prevalence of sweetpotato viruses in northern Uganda relative to central Uganda to the differences in rainfall distribution pattern [6]. Northern Uganda experiences longer dry periods with a unimodal pattern of rainfall compared to an even distribution of rainfall throughout the year for central Uganda [6]. In the three East African countries of Kenya, Uganda and Tanzania, cases of high sweetpotato virus incidence have consistently been reported in areas around Lake Victoria, which receive abundant rainfall throughout the year [6,8,13,15]. Such areas with regular rainfall have a continuous sweetpotato production pattern which maintains infected plants in the production system for long periods and favours continuous proliferation of whitefly vectors [8]. In contrast, the northern region has a prolonged dry spell during December–April, which scorches most vines and reduces their reuse and the multiplication of vectors of sweetpotato viruses. Reports indicate that uniform rainfall distribution within the Lake Victoria basin supports proliferation, abundance and distribution of the whitefly vector throughout the year [23,24]. Stable whitefly populations are maintained by continuous sweetpotato production and even distribution of rainfall throughout the year, which is not the case in northern Uganda.

## 5 Conclusion

The four sweetpotato viruses detected in the region are the major viruses reported to infect sweetpotato in other parts of Uganda and East Africa. The most frequently detected virus was SPFMV and least detected was SPCSV. The two viruses SPCSV and SPFMV are the most significant viruses of sweetpotato worldwide because co-infection of a plant results in a devastating disease syndrome, with associated yield losses in the range of 65–98%. Overall the study found low frequency of occurrence of the viruses in the Acholi sub-region, indicating a lower burden to sweetpotato production within this sub-region compared to previous studies conducted in central, western and eastern Uganda.

## Funding

This work was supported by PEARL grant [Investment ID: OPP1112536] from Bill and Melinda Gates Foundation.

## Conflict of interests

The authors declare that they have no competing interests

## References

[1] Karyeija R.F., Gibson R.W., Agricultural N., Valkonen J.P.T. (1998). The Significance of sweetpotato feathery mottle virus in subsistence sweetpotato production in Africa. Plant Dis..

[2] Low J.W., Arimond M., Osman N., Cunguara B., Zano F., Tschirley D. (2007). Ensuring the supply of and creating demand for a biofortified crop with a visible trait: lessons learned from the introduction of orange-fleshed sweet potato in drought-prone areas of Mozambique. Food Nutr. Bull..

[3] Kivuva B.M., Musembi F.J., Githiri S.M., Yencho C.G., Sibiya J. (2014). Assessment of production constraints and farmers’ preferences for sweetpotato genotypes. J. Plant Breed. Genet..

[4] Kim S.Y., Ryu C.H. (1995). Studies on the Nutritional Components of Purple Sweet Potato (Ipomoea batatas). 27.

[5] Ishida H., Suzuno H., Sugiyama N., Innami S., Tadokoro T., Maekawa A. (2000). Nutritive evaluation on chemical components of leaves, stalks and stems of sweet potatoes (Ipomoea batatas poir). Food Chem..

[6] Aritua V., Bua B., Barg E., Vetten H.J., Adipala E., Gibson R.W. (2007). Incidence of five viruses infecting sweetpotatoes in Uganda ; the first evidence of Sweet potato caulimo-like virus in Africa. Plant Pathol..

[7] Wasswa P., Otto B., Maruthi M.N., Mukasa S.B., Monger W., Gibson R.W. (2011). First identification of a sweet potato begomovirus (sweepovirus) in Uganda : characterization, detection and distribution. Plant Pathol..

[8] Mukasa S.B., Rubaihayo P.R., Valkonen J.P.T. (2003). Incidence of viruses and virus like diseases of sweetpotato in Uganda. Plant Dis..

[9] Adane A. (2010). Associated viruses threatening sweetpotato improvement and production in Ethiopia. Afr. Crop Sci. J..

[10] Domola M.J. (2003). Survey and Characterisation of Sweet Potato Viruses in South Africa.

[11] Aritua V., Gibson R., Vetten J., Anderson P.K., Morales FJ with the collaboration of Jones AL MR (2005). Serological Analysis of Sweetpotatoes Affected by Sweetpotato Virus Disease in East Africa.

[12] Aritua V., Barg E., Adipala E., Vetten H.J. (2007). Sequence analysis of the entire RNA genome of a sweet potato chlorotic fleck virus isolate reveals that it belongs to a distinct carlavirus species. Arch. Virol..

[13] Rukarwa R., Mashingaidze A., Kyamanywa S., Mukasa S. (2010). Detection and elimination of sweetpotato viruses. Afr. Crop Sci. J..

[14] Di Feo L., Nome S.F., Biderbost E., Fuentes S., Salazar L.F. (2000). Etiology of sweet potato chlorotic dwarf disease in Argentina. Plant Dis..

[15] Gibson R.W., Mpembe I., Alicai T., Carey E.E., Mwanga R.O.M., Seal S.E. (1998). Symptoms, aetiology and serological analysis of sweet potato virus disease in Uganda. Plant Pathol..

[16] Valverde R.A., Clark C.A., Valkonen J.P.T. (2007). Viruses and virus disease complexes of sweetpotato. Plant Viruses.

[17] Tairo F., Kullaya A., Valkonen J.P.T. (2004). Incidence of viruses infecting sweetpotato in Tanzania. Plant Dis..

[18] Tesfaye T., Feyissa T., Abraham A. (2011). Survey and Serological Detection of Sweet Potato (Ipomoea batatas (L.) Lam.) Viruses in Ethiopia.

[19] Opiyo S.A., Ateka E.M., Owuor P.O., Manguro L.O.A., Karuri H.W. (2010). Short communication survey of sweet potato viruses in western kenya and detection of cucumber mosaic virus. J. Plant Pathol..

[20] Kwak H.R., Kim M.K., Shin J.C., Lee Y.J., Seo J.K., Lee H.U. (2014). The current incidence of viral disease in Korean sweet potatoes and development of multiplex RT-PCR assays for simultaneous detection of eight sweet potato viruses. Plant Pathol. J..

[21] Masek T., Vopalensky V., Suchomelova P., Pospisek M. (2005). Denaturing RNA electrophoresis in TAE agarose gels. Anal. Biochem..

[22] Opiyo S.A., Ateka E.M., Owuor P.O., Manguro L.O., Miano D.W. (2010). Development of a multiplex pcr technique for simultaneous detection of sweet potato feathery mottle virus and sweet potato chlorotic stunt virus. J. Plant Pathol..

[23] Mukasa S.B., Rubaihayo P.R., Valkonen J.P.T. (2006). Interactions between a crinivirus, an ipomovirus and a potyvirus in coinfected sweetpotato plants. Plant Pathol..

[24] De Souza J., Cuellar W.J. (2011). Sequence Analysis of the Replicase Gene of “Sweet Potato Caulimo-like Virus” Suggests That This Virus is a Distinct Member of the Genus Cavemovirus.

[25] Alicai T., Fenby N.S., Gibson R.W., Adipala E., Vetten H.J., Foster G.D., Seal S.E. (1999). Occurrence of two serotypes of sweet potato chlorotic stunt virus in East Africa and their associated differences in coat protein and HSP70 homologue gene sequence. Plant Pathol..

